# Enhanced Electrocatalytic Activity by RGO/MWCNTs/NiO Counter Electrode for Dye-sensitized Solar Cells

**DOI:** 10.1007/s40820-015-0043-7

**Published:** 2015-05-17

**Authors:** Majid Raissan Al-bahrani, Waqar Ahmad, Hadja Fatima Mehnane, Ying Chen, Ze Cheng, Yihua Gao

**Affiliations:** 1 Center for Nanoscale Characterization & Devices, Wuhan National Laboratory for Optoelectronics, School of Physics, Huazhong University of Science and Technology, Wuhan, 430074 People’s Republic of China; 2 Faculty of Science, Thi-Qar University, Nasiriyah, Iraq; 3 School of Physics and Technology and Key Laboratory of Artificial Micro- and Nano-structure of Ministry of Education, Wuhan University, Wuhan, 430072 People’s Republic of China; 4 School of Physics, Huazhong University of Science and Technology, Wuhan, 430074 People’s Republic of China

**Keywords:** Graphene oxide, Carbon nanotubes, Nickel oxide, Counter electrode, Dye-sensitized solar cells

## Abstract

We applied the reduced graphene oxide/multi-walled carbon nanotubes/nickel oxide (RGO/MWCNTs/NiO) nanocomposite as the counter electrode (CE) in dye-sensitized solar cells (DSSCs) on fluorine-doped tin oxide substrates by blade doctor method. Power conversion efficiency (PCE) of 8.13 % was achieved for this DSSCs device, which is higher than that of DSSCs devices using NiO, RGO, and RGO/NiO-CE (PCE = 2.71 %, PCE = 6.77 % and PCE = 7.63 %). Also, the fill factor of the DSSCs devices using the RGO/MWCNTs/NiO-CE was better than that of other CEs. The electron transfer measurement of cyclic voltammetry and electrochemical impedance spectroscopy showed that RGO/MWCNTs/NiO film could provide fast electron transfer between the CE and the electrolyte, and high electrocatalytic activity for the reduction of triiodide in a CE based on RGO/MWCNTs/NiO in a DSSC.

## Introduct**i**on

In recent, the demand for alternative clean and sustainable energy technologies increases worldwide because of the pollution caused by fossil fuels and their advanced exhaustion. Dye-sensitized solar cells (DSSCs) which can convert the sun energy into electricity are believed to be a promising energy conversion technology. It becomes essential and very important to improve the DSSCs performance such as low production cost, low environmental impact during fabrication, and high energy conversion efficiency [1]. In the case of the original Grätzel design, the DSSC has three primary parts: photoanode, liquid electrolytes, and platinum (Pt) deposited on another transparent conducting oxide (TCO) substrate [1, 2]. The photoanode which determines the device efficiency is usually fabricated using TiO_2_ due to its large ratio of surface area to volume for dye materials [3–6], and the issues related to the counter electrode (CE) must also be addressed. As known, the CE in DSSCs can quickly transport electrons from the electrode substrate to the electrolyte and effectively catalyze the iodide–triiodide (I^−^/I_3_^−^) redox reaction in the electrolyte. However, for long-term stability and cost-effective construction of the DSSCs, Pt-CE suffers from its high price, rarity, and susceptibility to corrosion by iodide electrolyte. Using alternative materials of Pt in CE is expected to reduce fabrication cost of DSSCs. Nanocarbon, carbon black, hard carbon spherules, polymer, polymer/Pt, and polymer/carbon have been introduced as catalysts for DSSCs [7–17]. Carbonaceous materials are highly important materials in either their pristine or their composite forms due to their low cost and abundance [18–23]. Single-walled carbon nanotubes (SWCNTs), multi-walled carbon nanotubes (MWCNTs), multi-walled carbon nanotubes/graphene (MWCNTs/G), MWCNTs/polymers, and MWCNTs/Pt-CE in DSSCs have been considered as ideal alternative sources to Pt owing to their good properties such as high conductivity, large specific surface area, and chemical stability [24–29]. Recently, Wang et al. prepared a nickel oxide (NiO)-coated fluorine-doped tin oxide (FTO) glass CE in DSSCs, in which NiO was taken as a catalytic role towards I^−^/I_3_^−^ redox couple [30]. The conductive behaviors of metal oxide with CNTs (or graphene) as the CE have been investigated and an increase of the power conversion efficiencies was observed [31, 32]. Yeh et al. demonstrated that reduced graphene oxide (RGO) with good electrocatalytic ability for reducing I_3_^−^ is a promising catalyst for the CE of DSSCs [33]. However, hybrid materials such as graphene/cobalt sulfide [34] and RGO/Cu_2_S [35] have been reported to show improved catalytic activity and conductivity relative to single-component materials which enhanced efficiency in DSSCs. A RGO/MWCNTs/NiO nanocomposite would be an excellent candidate as counter electrode material for DSSCs.

In this work, we used the RGO/MWCNTs/NiO nanocomposite as a cathode material in DSSCs for catalyzing the I^−^/I_3_^−^ redox reaction and transporting electrons from the FTO to the electrolyte. The combination of high electrocatalytic of NiO and outstanding conductivity of graphene and MWCNTs showed superior performance. Cyclic voltammetry (CV) and electrochemical impedance spectroscopy (EIS) confirmed that the RGO/MWCNTs/NiO-CE has electrocatalytic ability to reduce I_3_^−^, and the charge-transfer resistance (*R*_ct_) was lower. Due to the high catalytic activity and the superior electrical conductivity, the RGO/MWCNTs/NiO-CE also showed excellent photovoltaic performance.

## Experimental

### Chemicals and Materials

In this work, the materials and solvents were purchased from Sinopharm Chemical Reagent Co. MWCNTs and graphene oxide (GO) were bought from Beijing BoyuGaoke New Material Technology Co. TiO_2_ paste and ruthenium 535-bis-TBA (N719) were purchased from Solaronix. The electrolyte was produced by a solution of 0.05 M I_2_, 0.1 M LiI (Adamas-beta), 0.6 M 1-methyl-3-butylimidazolium iodide (TCl), 0.1 m guanidinium thiocyanate (TCl), and 0.5 M 4-tert-butylpyridine (TCl) mixed in 3-methoxypropionitrile solution (Alfa Aesar). Millipore water (18.25 MΩ cm) was used in the whole process. FTO glass with a sheet resistance of 8 Ω/square and a thickness of 2.2 mm, supplied by Nippon Sheet Glass, was used for both electrodes.

### Preparation of Counter Electrode

**Fig. 1:**
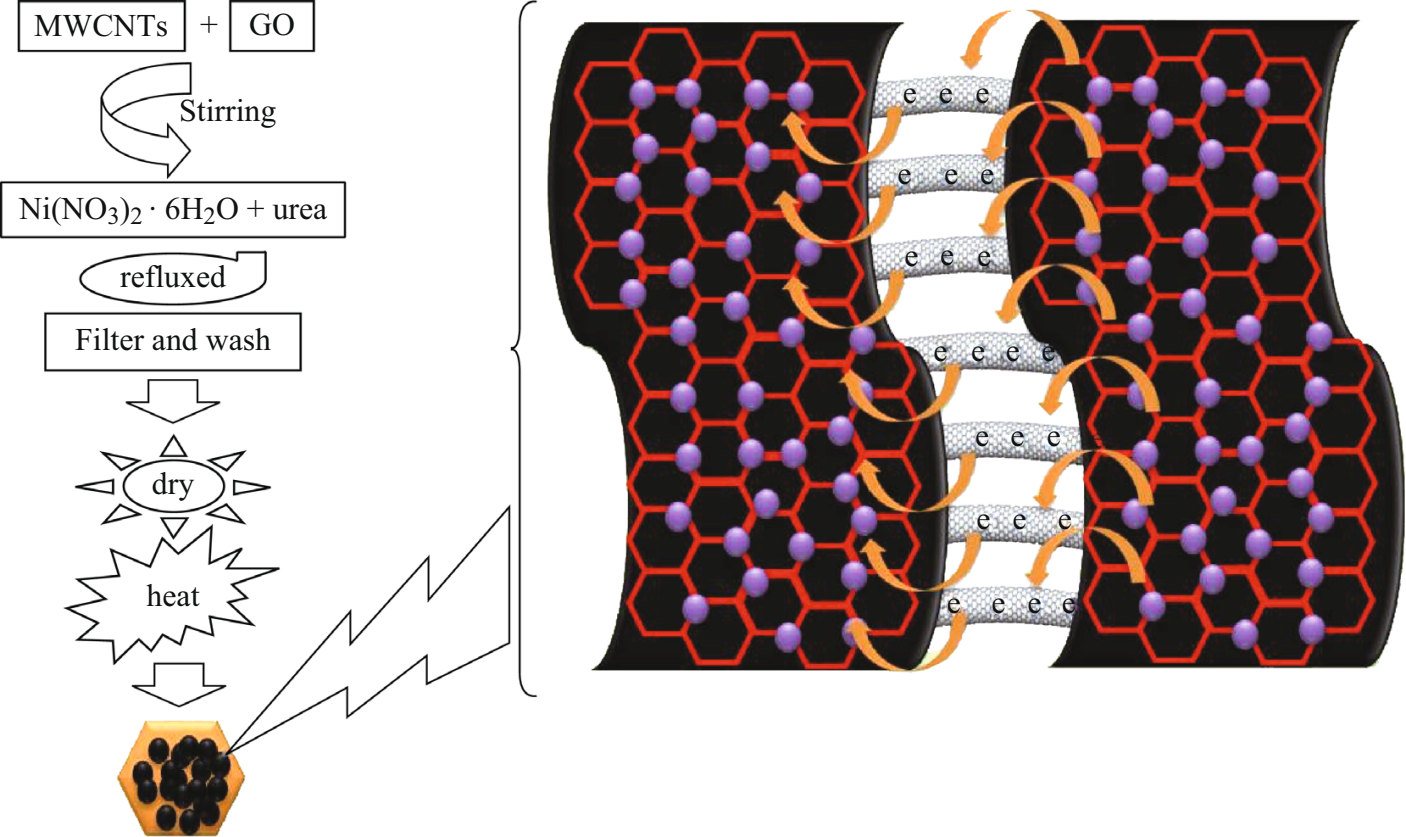
Schematic flowchart showing the fabrication process for RGO/MWCNTs/NiO composite

CE for the DSSCs was prepared on coated FTO glass substrate. Firstly, glass substrates coated with FTO were washed with detergent solution and rinsed with deionized (DI) water, and then cleaned in an ultrasonic bath for 15 min in the end rinsed with ethanol and dried in air. Before that, the RGO/MWCNTs/NiO composite was prepared in the following steps as illustrated in Fig. 1. MWCNTs (0.02 g) were refluxed with HNO_3_ at 80 °C for 6 h and GO (80 mL) suspension with a concentration of 1 mg mL^−1^ under strong stirring. At room temperature, 50 mL aqueous solution containing 0.4362 g nickel (II) nitrate hexahydrate (Ni(NO_3_)_2_·6H_2_O) and 1.5 g urea was slowly dropped into the GO and CNTs suspension with stirring for 30 min. Then, the mixture was refluxed at 100 °C for 12 h in an oil bath. The reaction product was filtered and washed with DI water and ethanol successively several times. Finally, it was dried at 60 °C for 24 h and then heat treated at 250 °C for 2 h in air. To prepare the RGO/MWCNTs/NiO paste, 1 g RGO/MWCNTs/NiO powder was mixed with 0.5 g ethyl cellulose in 8 mL ethanol. Then 0.2 mL acetic acid and 3 g terpineol were slowly added with continuous mixing for 36 h. To prepare the RGO/MWCNTs/NiO-CE, the RGO/MWCNTs/NiO paste was coated on the FTO glass substrate by the doctor-blade method. Then the formed films were annealed at 400 °C for 30 min using a muffle furnace. For comparison, RGO and RGO/NiO were also prepared under the same synthesis conditions.

### DSSC Device Fabrication

**Fig. 2:**
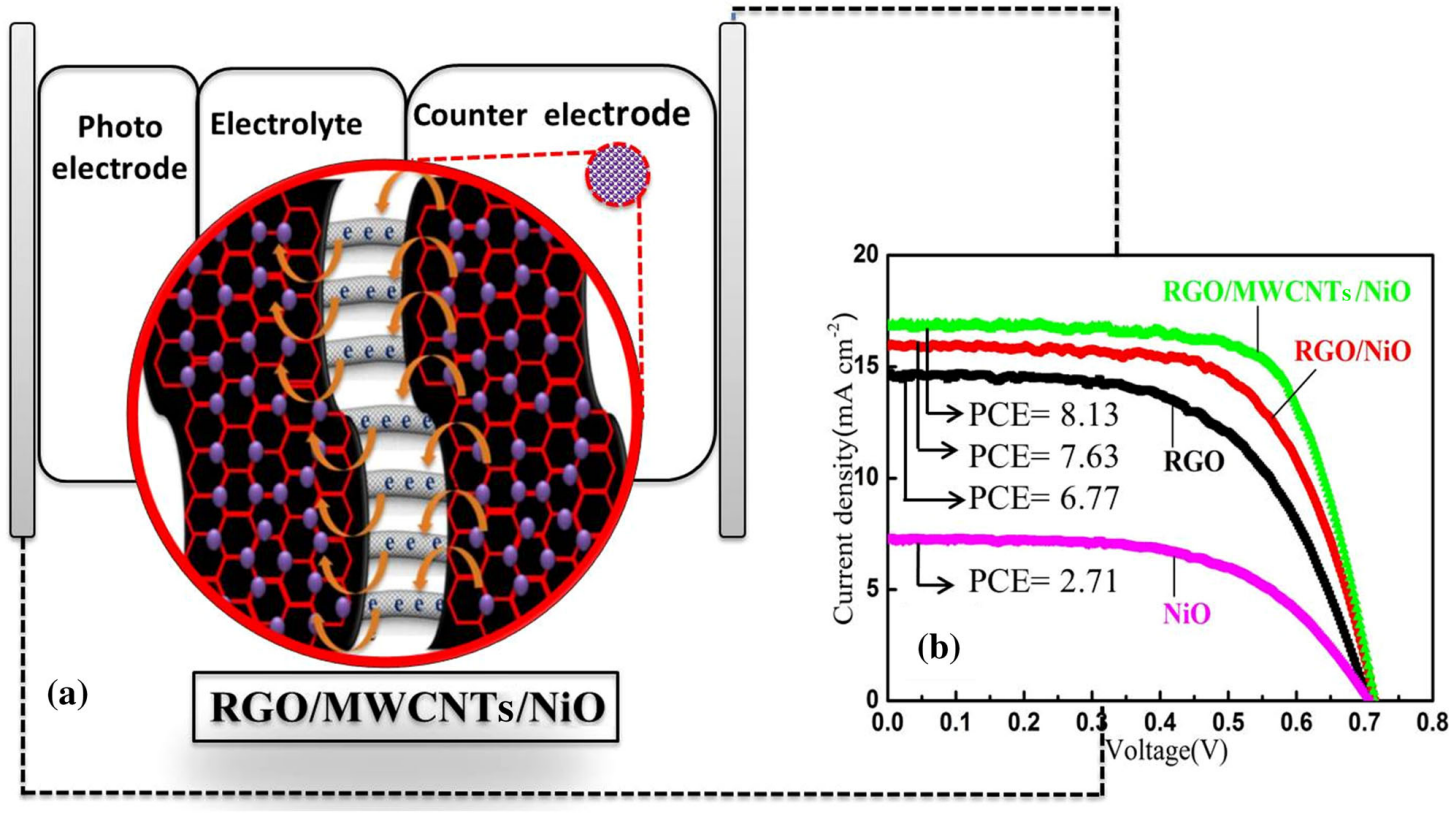
**a** Schematic illustration of a DSSCs device using RGO/MWCNTs/NiO-CE **b** The current–voltage characteristic curves of DSSC fabricated with different CEs

For synthesis of the photoanode, TiO_2_ layer-by-layer hierarchical nanosheets (TiO_2_ LHNs) and their paste were fabricated according to the previously reported method [36, 37]. Briefly, the TiO_2_ LHNs powder (0.9 g) was added to the solution containing ethanol/DI water (4:1, volume) and acetylacetone (0.16 mL) for a 3 h stir. After that, TiO_2_ paste was applied on pre-treated FTO glass by the doctor-blade method and sintered at 500 °C for 30 min to achieve crystallization in a muffle furnace. The TiO_2_ electrode was kept in a dye sensitization (0.5 mM in a mixture of 1:1 acetonitrile/tert-butanol) for at least 12 h at 60 °C in a sealed beaker, then rinsed with ethanol, and dried under nitrogen flow. The TiO_2_ photoanode was assembled with the CE manufactured as in Fig. 2a. The electrolyte solution was inserted through holes drilled in the CE, and the holes were sealed with hot-melt film and a cover glass finally.

### Measurements of DSSCs

The morphologies and structures of the samples were characterized using high-resolution field emission scanning electron microscopy (SEM, FEI Nova Nano-SEM 450), transmission electron microscopy (TEM, Tecnai G2 20 U-Twin), and X-ray diffractometer (XRD, PANalytical B.V. X’Pert PRO). The redox properties of dye were examined by cyclic voltammetry at a scan rate of 50 mV s^−1^. The electrolyte solution was an acetonitrile solution containing 10 mM LiI, 5 mM I_2_, and 0.1 M LiClO_4_. Tests were conducted in a three-electrode one-compartment cell, where CE, Pt, and Ag/AgCl were taken as the working electrode, auxiliary electrode, and reference electrode, respectively. The photovoltaic current density–voltage (*J*–*V*) characteristics of the prepared DSSCs were measured under illumination conditions (100 mW cm^−2^, AM 1.5), which was verified using Si photodiode, solar-simulator illumination (Newport, USA) on the active cell area of 0.15 cm^2^. The light-to-electric power conversion efficiency (PCE) and fill factor (FF) were calculated according to the equations [38]:

1 $$ {\text{FF}}\,=\, \frac{{V_{\hbox{max} } \times J_{\hbox{max} } }}{{V_{\text{oc}} \times J_{\text{sc}} }} $$

2 $$ {\text{PCE}} = \frac{{V_{\hbox{max} } \times J_{\hbox{max} } }}{{P_{\text{in}} }} \times 100\;\% = \frac{{V_{\text{oc}} \times J_{\text{sc}} \times FF}}{{P_{\text{in}} }} \times 100\;\% $$

where *V*_max_ and *J*_max_ are, respectively, the voltage and the current density under the maximum power output in the *J*–*V* curves, *J*_SC_ is the short-circuit current density (mA cm^−2^), *V*_OC_ is the open-circuit voltage (V), and *P*_in_ is the incident light power. EIS was examined at the open-circuit potential under the same illumination condition as the measurement of the *J*–*V* curves. The data were obtained by using Z-view software NOVA 1.7 to analyze the results from Auto-lab electrochemical work station (model AUT84315, the Netherlands) [39].

## Results and Discussion

**Fig. 3:**
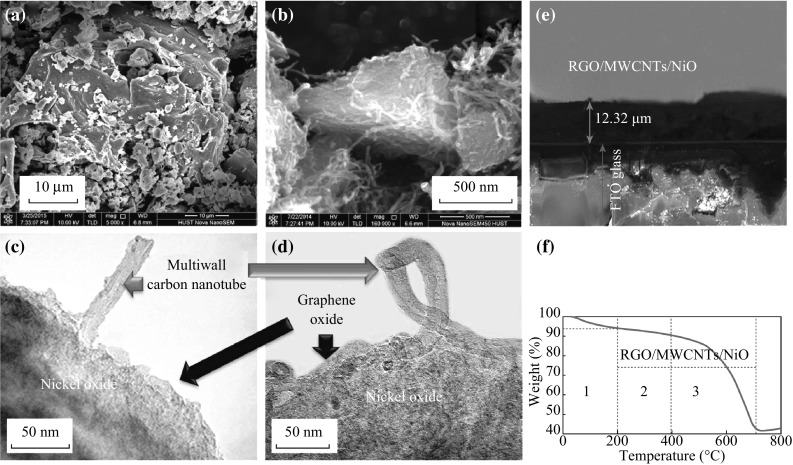
**a**–**d** SEM and TEM images of RGO/MWCNTs/NiO composite. **e** SEM image of RGO/MWCNTs/NiO film on FTO glass substrate (cross section), showing the thickness of the film. **f** The TGA analysis of the RGO/MWCNTs/NiO

Figure 3 shows SEM and TEM images of RGO/MWCNTs/NiO nanocomposites. NiO nanoparticles were anchored on the surface of RGO sheets, which were separated by MWCNTs with less aggregation. From TEM images, NiO nanoparticles with uniform size were distributed on the surface of the RGO and connected by MWCNTs to form continuous network (Fig. 3c, d). More active sites were available in this structure, and electron transport properties as well as the cell performance are expected to be improved.

Thermo gravimetric analysis (TGA) measurements were carried out to determine the mass ratio of RGO/MWCNTs/NiO composites (Fig. 3f). The first step occurred around ~200 °C, which was due to the removal of the physisorbed water. The large weight loss below ~400 °C was attributed to the removal of RGO from the composites. Between 400 and 710 °C, the graphitic carbon burnt off accounting for the second burn stage. Above 710 °C, the TGA trace was stable with no further weight loss and only NiO remained.

**Fig. 4:**
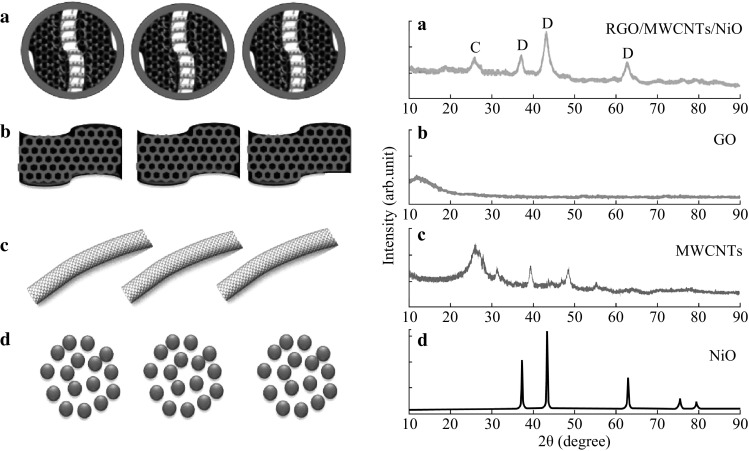
XRD patterns of GO-, NiO-, MWCNTs-, and RGO/MWCNTs/NiO-CE

XRD results of samples are shown in Fig. 4. For MWCNTs, it shows a characteristic diffraction peak at 2*θ* of 26° (0 0 2), whereas RGO/MWCNTs/NiO nanocomposite shows new diffraction peaks at 37.2° (1 1 1), 42.8° (2 0 0), and 62.4° (2 2 0) which ascribe to the crystal structure of NiO nanoparticles. However, no characteristic diffraction peak of GO observed in the RGO/MWCNTs/NiO nanocomposite indicated the successful reduction of GO to RGO.

**Fig. 5:**
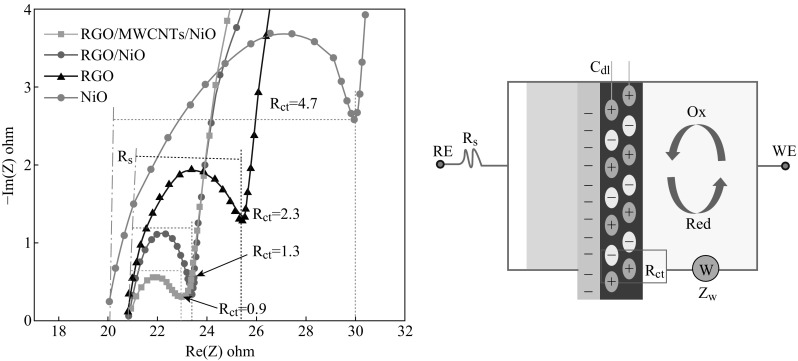
EIS analysis of RGO-, RGO/NiO-, and RGO/MWCNTs/NiO-CE and equivalent circuit models. (*R*_s_, *R*_ct_, *C*_dl_, and *Z*_w_ are serial resistance, charge-transfer resistance of electrode, double layer capacitance, and diffusion impedance, respectively)

To study the electrochemical behavior of composites, EIS was conducted under illumination of AM 1.5 G (100 mW cm^−2^) and a potential amplitude of 10 mV with frequencies of 10 mHz–100 kHz to understand the effect of the electrocatalytic activities of different CE on the I_3_^−^ reduction. The impedance spectra were illustrated as Nyquist plots and the equivalent circuit (Fig. 5). These works focus on the semicircle in the highest frequency region describing the electron transport at the CE/electrolyte interface. The charge-transfer resistance (*R*_ct_) occurs at the contact interface between the electrode and the electrolyte [40]. *R*_s_ is the series resistance including the TCO’s sheet resistance and the cell’s contact resistances as itemized in Table 1. It can be seen that RGO/MWCNTs/NiO-CE has the smallest diameter of semicircle which was related to the less *R*_ct_ (0.9 Ω), whereas RGO and NiO-CE have the largest *R*_ct_ (2.3 and 4.7 Ω). When NiO nanoparticles were adopted with RGO, the *R*_ct_ decreased to 1.3 Ω and the DSSC device with the RGO/MWCNTs/NiO-CE exhibits the smallest *R*_ct_, indicating the optimal compositions of RGO/NiO and MWCNTs. Since *R*_ct_ of the CE affects the FF and PCE of DSSCs in a negative way [41–43], indicating the improved electrocatalytic activity for redox electrolyte and high electron transfer kinetics, it will lead to a greater diffusion of the iodide/triiodide (I^−^/I_3_^−^) from the bulk solution to the electrode surface.

**Table 1:** Photovoltaic performance of RGO-, RGO/NiO-, and RGO/MWCNTs/NiO-CE based DSSCs prepared by doctor-blade method

Counter electrode	*J*_sc_ (mA cm^−2^)	*V*_oc_ (V)	FF	PCE (%)	*R*_s_ (Ω)	*R*_ct_ (Ω)
NiO	7.31	0.70	0.53	2.71	20.02	4.7
RGO	14.36	0.70	0.67	6.77	20.67	2.3
RGO/NiO	15.86	0.71	0.67	7.63	20.71	1.3
RGO/MWCNTs/NiO	16.80	0.71	0.68	8.13	20.62	0.9

**Fig. 6:**
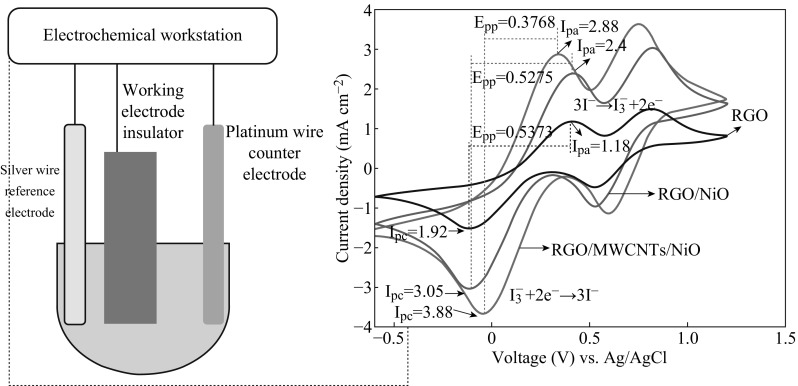
Cyclic voltammograms for RGO, RGO/NiO, and RGO/MWCNTs/NiO electrodes. The electrolyte was acetonitrile solution containing 10 mM LiI, 5 mM I_2_, and 0.1 M LiClO_4_. Pt electrode was used as auxiliary electrode and Ag/AgCl works as reference electrode. The scan rate is 50 mV s^−1^

In order to understand further the improved DSSC devices with RGO/MWCNTs/NiO-CE, we measured CV curves of the I^−^/I_3_^−^ redox couple on the RGO/MWCNTs/NiO, RGO/NiO and RGO CE, respectively. From the results shown in Fig. 6, two sets of peaks were observed, which is due to the redox reaction of the I^−^/I_3_^−^ redox shuttle and another redox reaction of the I_3_^−^/I_2_^−^ redox couple [44]. It is well known that two key parameters for estimating the catalytic activities of the CE are the peak-to-peak separation (*E*_PP_) and peak current density (*I*_P_) [45]. Therefore, the magnitude of *I*_P_ is proportional to the ability of the CE to reduce the I_3_^−^ species, while the magnitude of *E*_PP_ is inversely proportional to the ability of the CE to reduce the I_3_^−^ species. The CE in the DSSC is responsible for catalyzing the regeneration of I^−^ from I_3_^−^ (I_3_^−^ + 2e^−^→3I^−^) [46]. The RGO/MWCNTs/NiO electrode shows larger *I*_P_ (2.88 mA) from the redox reaction of the I^−^/I_3_^−^ redox shuttle than the other two electrodes with the RGO and RGO/NiO electrodes showing *I*_P_ of 1.18 and 2.4 mA, respectively (Table 2). In addition, *E*_pp_ of RGO/MWCNTs/NiO-CE was lower than those of the RGO and RGO/NiO-CE. The RGO/MWCNTs/NiO-CE offers a high enough active area for faster and stronger redox reactions and a higher electrocatalytic effect for the reduction of I_3_^−^ at the RGO/MWCNTs/NiO electrode.

**Table 2:** *E*_pp_, *I*_pa_, and *I*_pc_ of RGO, RGO/NiO and RGO/MWCNTs/NiO electrodes

Counter electrode	*E*_pp_	*I*_pa_ (mA cm^−2^)	*I*_pc_ (mA cm^−2^)
RGO	0.53	1.18	1.92
RGO/NiO	0.52	2.4	3.05
RGO/MWCNTs/NiO	0.37	2.88	3.88

The *J*–*V* curves of photovoltaic performance for DSSCs devices with NiO, RGO, RGO/NiO, and RGO/MWCNTs/NiO different CEs are shown in Fig. 2b. The devices' performance parameters including *J*_sc_, *V*_oc_, FF, and PCE are summarized in Table 1. The PCE and FF were calculated according to the Eqs. (1) and (2). The DSSC devices with RGO/MWCNTs/NiO-CE reach the highest power conversion efficiency. The PCE significantly enhanced from 6.77 % for RGO CE cell to 8.13 % for RGO/MWCNTs/NiO-CE one. This may be due to the increase of electrocatalytic activity toward I^−^/I_3_^−^ redox species and decrease of *R*_ct_. From CV curves in Fig. 6 and values of *J*_sc_ and PCE in Table 1, the DSSC devices with RGO/MWCNTs/NiO-CE exhibit the best photovoltaic performances, as well as better FF compared with other CEs. The enhanced *J*_sc_ maybe results from the enhanced diffusivity of I^−^/I_3_^−^ redox species within CE [47]. However, the improved performance should attribute to the incorporation of MWCNTs into RGO/NiO which provides larger space allowing easy diffusion between the redox species.

**Fig. 7:**
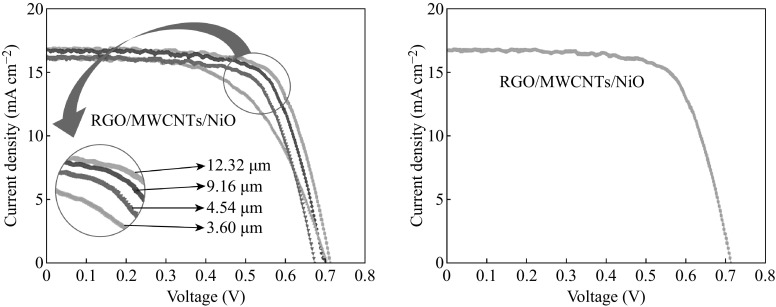
The current–voltage curves of DSSCs using different thickness of RGO/MWCNTs/NiO film as counter electrodes

RGO/MWCNTs/NiO films with different thicknesses of 3.6–12.7 μm were prepared to investigate the film thickness effect on performances of DSSCs (Table 3). As shown in Fig. 7, *V*_oc_ and FF increase with the film thickness, whereas *J*_sc_ is almost unchangeable. The highest photovoltaic efficiency of 8.13 % was observed in 12.7-μm DSSC (SEM image of the cross section shown in Fig. 3e). The RCT between electrolyte and RGO/MWCNTs/NiO increases with decreasing the film thickness, leading to the decrease of the FF and the PCE of DSSCs [48]. This is due to the insufficient catalytic activity for the reduction of triiodide of the thinner RGO/MWCNTs/NiO layers.

**Table 3:** Photoelectric performances of the DSSCs using various thickness of RGO/MWCNTs/NiO film as CE

Thickness (μm)	*J*_sc_ (mA cm^−2^)	*V*_oc_ (V)	FF	PCE (%)
12.32	16.80	0.71	0.68	8.13
9.16	16.69	0.69	0.64	7.37
4.54	16.18	0.67	0.63	6.82
3.60	16.05	0.70	0.58	6.51

In addition, it should be mentioned that decrease of NiO particle size will enhance the conductivity of RGO/MWCNTs/NiO composites because of the synergistic effect between RGO and MWCNTs. CNTs not only prevent aggregation of RGO/NiO but also improve the electron transport properties of RGO/MWCNTs/NiO composite owing to their special conductivity. Moreover, the restricting effect of RGO makes NiO nanoparticles provide more active sites. It is because of the unique structure and properties, RGO/MWCNTs/NiO composite has enhanced electrochemical performance compared with that of RGO/NiO and MWCNTs/NiO ones.

## Conclusion

In this paper, we fabricated RGO/MWCNTs/NiO composite and applied it in DSSC as a CE by blade doctor method. High PCE of 8.13 % was achieved in such DSSC, which is much higher than that of NiO (2.71 %), RGO (6.77 %) and RGO/NiO (7.63 %). Also, it was found that the RGO/MWCNTs/NiO-CE has less charge-transfer resistance at the electrolyte/CE interface and higher catalytic activity for reduction of I_3_^−^ to I^−^. The improved performances maybe attribute to the enhanced electrode conductivity, the increased effective interfacial area between RGO/MWCNTs/NiO and electrolyte, as well as the contact area between RGO/NiO and other materials by MWCNTs.
